# Neuroglial markers of damage in autoimmune neurology

**DOI:** 10.1177/17562864261433329

**Published:** 2026-04-03

**Authors:** Alessandro Dinoto, Sara Mariotto

**Affiliations:** Neurology IV—Neuroimmunology and Neuromuscular Diseases, Fondazione IRCCS Istituto Neurologico Carlo Besta, Via Celoria 11, Milan 20133, Italy; Neurology Unit, Department of Neurosciences, Biomedicine and Movement Sciences, University of Verona, Verona, Italy

**Keywords:** autoimmune encephalitis, biomarker, glial fibrillary acid protein, myelin oligodendrocyte glycoprotein antibody-associated disease, neurofilament light chain, neuromyelitis optica, paraneoplastic neurological syndrome

## Abstract

The advancements in assays for the detection of neuroglial markers of damage, such as neurofilament light chain and glial fibrillary acid protein, have led to a growing interest in their investigation in neurological disorders. In this review, we aim to summarize evidence regarding the role of biomarkers of neuroglial damage in autoimmune neurological disorders of the central nervous system, including myelin oligodendrocyte glycoprotein antibody-associated disease, neuromyelitis optica spectrum disorder, autoimmune encephalitis, and paraneoplastic neurological syndromes. We will discuss how these biomarkers can help our understanding of disease mechanisms and how these results can be potentially translated into clinical practice, helping clinicians in the differential diagnosis, in prognostication, and prediction of the risk of relapse.

## Introduction

In recent years, the improvement of diagnostic assays for the detection of markers of neuroglial damage, such as neurofilament light chain (NfL) or glial fibrillary acid protein (GFAP), have allowed these proteins to be routinely detected in serum or plasma samples, and not only in the cerebrospinal fluid (CSF), as for older generation enzyme-linked immunoassays (ELISA).^1,2^

These advancements have led biomarker investigation in easily accessible samples, leading to further studies of neurological disorders in which CSF analysis is not routinely performed, and also allowing longitudinal evaluation of samples.

Among inflammatory neurologic disorders, neuroglial biomarkers have been widely investigated in multiple sclerosis (MS), but recently, a growing body of evidence has supported their potential role also in less frequent autoimmune disorders of the central nervous system (CNS).^3^ Biomarkers of neuroglial damage may have a potential role in clinical practice, including helping clinicians in the differential diagnosis and predicting disease course or relapses.

The aim of this review is to provide an updated overview of neuroglial markers of damage in the field of rare autoimmune disorders of the CNS. The discussion of biomarkers in MS, the most common CNS neuroimmune disorder, goes beyond the scope of this review and thus will not be included, even though MS will be mentioned in the manuscript for comparative purposes.

## Myelin oligodendrocyte glycoprotein antibody-associated disease and neuromyelitis optica spectrum disorder

Myelin oligodendrocyte glycoprotein antibody-associated disease (MOGAD) and neuromyelitis optica spectrum disorder (NMOSD) emerged as distinct CNS inflammatory disorders, with unique clinico-radiological, immunological, and pathological characteristics. As a consequence, the differential diagnosis is of utmost importance to administer proper treatment strategies.^4^

MOGAD, as an incidence of ≈3–4 million-person-years, affects both sexes almost equally, and both children and adults, with approximately 30% of patients presenting below the age of 18 years and 25% over 50 years.^5^ The most common clinical manifestations are optic neuritis, myelitis, acute disseminated encephalomyelitis (ADEM), brainstem/cerebellar involvement, and cortical encephalitis.^6,7^ Diagnosis is based on the presence of serum/CSF MOG antibodies (MOG-Abs) and a compatible clinical/radiological phenotype.^8^ A relapsing disease course is reported in 40%–80% of MOGAD, and many patients show a favorable recovery, with 10% experiencing a poor outcome.^9,10^ Disease course and final outcome are highly unpredictable. Acute treatment strategies include intravenous steroids and intravenous immunoglobulins (IvIg)/plasma exchange. The use of long-term immunosuppressive treatment in MOGAD is still a matter of debate, as a relapsing course is observed in about half of cases; thus, the benefits and risks of immunosuppressants should be carefully evaluated in each case.^11^ Immunosuppressive treatment can be proposed in patients with incomplete recovery from the onset of an attack, clinically severe/life-threatening first attack, or evidence of a relapsing disease course, and includes among the more common off-label options oral immunosuppressants (azathioprine, mycophenolate mofetil), tocilizumab, monthly IvIg, and rituximab. Trials with satralizumab, rozanolixizumab, azathioprine, and CAR-T treatment are currently ongoing.^6^

NMOSD is a rare CNS disorder with the highest estimated incidence/prevalence in the Afro-Caribbean region and Asia, and the lowest in Western countries and Australia/New Zealand.^12^ NMOSD is more common in females (9:1) and peaks in middle-aged adults, with rare occurrence in children.^13^ The main clinical manifestations include optic nerve, spinal cord, and/or are postrema involvement with compatible radiological involvement. Although a subgroup of patients is antibody negative, the diagnosis of NMOSD is based on the detection of antibodies to the water channel aquaporin-4 (AQP4-Abs), in serum.^14^ NMOSD is a severe condition with a high risk of disability and a relapsing disease course.^15^ Acute treatment includes intravenous steroids and plasma exchange/IvIg, whose prompt administration is of utmost importance to reduce final disability.^16^ Due to the frequently relapsing nature of the disease (about 90% of cases),^17^ chronic immunosuppression is always recommended.^18^ Long-term oral corticosteroids and/or steroid-sparing agents such as azathioprine, mycophenolate mofetil, and methotrexate have been used in NMOSD and are still employed in many countries as first-line agents.^19^ B-cell depleting agents, in particular rituximab, are also widely used in clinical practice as first-line treatments, and their role has been further supported by a recent clinical trial.^20^ In recent times, clinical trials have shown the efficacy of drugs targeting disease mechanisms of NMOSD, such as complement inhibition (eculizumab/ravulizumab), anti-IL6 receptor blockade (satralizumab), and depletion of anti-CD19-positive cells (inebilizumab), leading to their approval by regulatory agencies.^21–25^ In contrast to MOGAD,^26,27^ where neoplasms are rarely encountered, AQP4 + NMOSD may have a paraneoplastic origin, in particular in male patients presenting with longitudinally extensive myelitis in association with adenocarcinomas.^26,27^

## Biomarkers in MOGAD

The extent of axonal and glial damage in MOGAD has been investigated in several studies, but remains unclear.^28^ Increased serum NfL has been reported in MOGAD, with higher levels during attacks and fluctuating over the 3 months following relapse, usually normalizing within 6 months.^29–34^ Some contradictory findings exist, with similar NfL levels in relapses and remission phases despite a correlation with disability at the time of sampling.^35^ NfL correlates with attack severity, but some studies report that it does not predict disease course or correlate with clinical/radiological phenotypes, though one study found higher levels in patients with brain MRI abnormalities and seizures.^32,36^ Longitudinal studies suggest NfL levels remain stable or decline over time in MOGAD, indicating that axonal injury mainly occurs at disease onset.^37^ A recent study showed that NfL decreases over time, is associated with disability at attack and radiological activity, independently predicts relapse risk (with higher concentration predicting shorter time to second relapse), and longitudinal changes forecast clinical recovery (with lowering concentrations during follow-up predicting an EDSS score <3 in monophasic patients). This highlights the potential value of monitoring NfL for predicting relapses and recovery. Treatment did not appear to affect NfL levels, and increases independent of clinical events were not observed.^38^

Data on GFAP are inconsistent: GFAP is typically lower in MOGAD compared to NMOSD, with some reports showing stable levels during relapse and remission without correlation to disability,^39^ while others observed increased GFAP during relapses correlating with disability.^30,33,35^ The serum NfL/GFAP ratio has been proposed as a useful marker to distinguish NMOSD, MOGAD, and MS.^33^ A recent study found that GFAP levels decrease over time, correlate with disease severity, and may predict clinical recovery. GFAP increase at the first attack seems to be minor compared to NfL peak, with less pronounced fluctuations over time and early normalization.^38^ Neither NfL nor GFAP increases during inter-attack periods, suggesting no attack-independent axonal or glial injury.^30^

Serum NfL is also elevated in pediatric MOGAD cases, particularly in those presenting with ADEM-like symptoms, encephalopathy, or longitudinally extensive transverse myelitis.^40,41^ NfL rises during clinical or radiological disease activity in children (within 20 days of onset), supporting its role as a biomarker for disease activity.^38^ While NfL alone does not predict relapsing disease, the ratio of NfL-to-MOG antibodies is significantly lower in relapsing than in non-relapsing pediatric cases, suggesting its potential utility for predicting disease course in this population.^42^

Of note, a recently published systematic review and meta-analysis report that neither NfL nor GFAP correlates with disease activity at sampling, although the heterogeneity of the included study might have influenced the results.^43^

## Biomarkers in NMOSD with AQP4-Abs

Markers indicating damage to axons and glial cells have demonstrated notable associations with disability and disease activity in NMOSD.^29^ Earlier research reported elevated CSF GFAP levels during relapses, which were higher than those detected in MS. CSF GFAP levels tend to decline following treatment and are linked with disability both at the time of attacks and over the follow-up.^44^ CSF NfL concentrations are also increased in NMOSD and correspond with disease activity. Some studies reported comparable NfL levels between NMOSD and MS, while others detected higher levels in NMOSD.^45,46^ These studies mostly employed first-generation ELISAs for CSF biomarker detection, as these early assays could not reliably identify the lower biomarker concentrations present in serum. This limited the translation into clinical practice of biomarkers and did not allow longitudinal studies.

More recently, advanced sensitive methods have facilitated the measurement of these biomarkers in serum, providing a useful tool for longitudinal monitoring. The strong correlation between CSF and serum levels further supports serum as a more accessible sample source.^47^ Both serum NfL and GFAP generally rise in NMOSD patients, with GFAP showing particularly high elevations, likely reflecting predominant astrocyte involvement. A limited number of studies reported similar GFAP levels in NMOSD patients and controls, primarily involving clinically stable cases.^39^ This suggests that GFAP may help predict AQP4 antibody positivity and assist in distinguishing NMOSD from MS. The serum GFAP/NfL ratio during relapse has been shown to be useful for differentiating MS from NMOSD, with higher values achieving diagnostic sensitivity of 73% and specificity of 75.8%.^29,32,33,47–50^ NfL levels also support differentiation between inflammatory and vascular conditions, with higher levels and more common in the latter.^51^

During NMOSD attacks, NfL and GFAP levels rise and correlate with disability measured by EDSS at the time of attacks, with GFAP showing the most consistent association. Recently, *Z*-score cutoffs of 3.0 for GFAP and 2.1 for NfL have been established to distinguish attack phases from remission in NMOSD.^52^ However, some evidence indicates that NfL levels do not predict recovery post-attack, and neither NfL nor GFAP reliably forecasts future relapses.^47,52–56^ Importantly, data from the N-Momentum trial analyzing inebilizumab’s effect in NMOSD showed that NfL strongly correlates with disability during attacks and predicts worsening disability post-attack, whereas GFAP levels can predict future attacks.^57,58^ Moreover, serum NfL, but not GFAP, may be useful for predicting spinal cord atrophy in NMOSD.^59^

Treatment with rituximab appears to influence biomarker levels, preventing significant serum GFAP elevation and reducing occurrences of subclinical GFAP increases.^53,54^ Tocilizumab similarly lowers NfL and GFAP concentrations, supporting the potential use of these biomarkers in monitoring therapeutic response.^56^ According to findings from the NMomentum trial, inebilizumab also decreases GFAP and NfL levels compared to placebo during attacks and over time.^57,58^

The dynamics of biomarker levels should be considered when interpreting NfL and GFAP in NMOSD patients, as GFAP peaks within 1 week of an attack and rapidly declines over the following 12 weeks, whereas NfL peaks approximately 5 weeks post-attack and decreases more gradually over 20 weeks.^52^ Increases in biomarker levels have also been reported in the week prior to relapse.^57,58^ In addition to disease phase and timing, factors such as age, BMI, coexisting conditions (which may affect biomarker clearance), and sex (particularly for serum GFAP) need to be considered.^52,60,61^ Age is also a relevant factor that influences serum NfL and GFAP levels.^50,62^

Lastly, recent evidence suggests that increased CSF concentrations of 14-3-3 protein can distinguish AQP4 + NMOSD from MOGAD and MS and track with disease status, but further independent validation of these results is warranted.^63^

## Biomarkers in double seronegative NMOSD

Double seronegative NMOSD remains a poorly understood condition with overlapping clinical-radiological features with AQP4 + NMOSD, but with scant evidence supporting shared disease mechanisms. As an example, IL6 blockade is an effective treatment for AQP4 + NMOSD, but no clear benefit was observed in double seronegative cases, implying heterogeneity in disease mechanisms.^25^ So far, limited studies have examined biomarker profiles in seronegative NMOSD. CSF levels tend to be lower in seronegative patients compared to those positive for AQP4 antibodies, with very high GFAP levels (>2050 ng/L) during relapse reported only in seropositive cases.^64,65^ However, some seronegative cases show increased GFAP, implying a possible unknown antibody targeting astrocytes and causing astrocytic damage.^66^ Recent serum studies confirmed significantly higher GFAP concentrations in seropositive (median 308.3 pg/mL) versus seronegative (median 103.4 pg/mL) NMOSD patients. Interestingly, two seronegative patients exhibited high GFAP, supporting astrocytic injury in a subset of seronegative NMOSD.^67^ NfL levels did not differ between seropositive and seronegative patients and, notably, did not correlate with disability in seronegative patients.^67^

## Autoimmune encephalitis and paraneoplastic neurological syndromes

Autoimmune encephalitis (AE) is characterized by an abnormal immune response against neuronal and glial proteins, leading to autoimmunity. Although infrequent in terms of epidemiology,^68^ AE is one of the most frequent causes of rapidly progressive cognitive impairment and represents a potentially treatable condition.^69,70^ Different clinical syndromes fall under the umbrella term of AE, including limbic encephalitis, extra-limbic encephalitis, brainstem encephalitis, and immune-mediated cerebellar ataxia. These syndromes have heterogeneous clinical manifestations, depending on the site of inflammation and specific associated autoantibody signatures.^71^

On a biological standpoint, AE can be associated with antibodies targeting neuronal surface or intracellular proteins. Antibodies targeting neuronal surface antigens can bind their target in vivo and are the pathogenic cause of the disease through cross-linking and antigen internalization, functional blocking of the cognate receptor, and blocking of protein–protein interactions.^72^

On the contrary, antibodies targeting intracellular proteins cannot access their targets in vivo and lack a pathogenic potential. Intracellular antigens are often shared between the nervous system and an underlying tumor, leading to an immune response against cancer cells and neurons. This immune response is mainly mediated by cytotoxic CD8+ T cells, which are responsible for early neuronal loss. The double-edge immune response against cancer and neuronal tissue defines paraneoplastic neurological syndromes (PNS).^73^

A third group of antibodies, including glutamic acid decarboxylase isoform 65 (GAD-65) and amphiphysin, shares common features with both antibodies against neuronal and intracellular targets, as the antigens are usually located intracellularly, but can be transiently expressed on the cell surface, implying a pathogenic potential.^74^

The different disease mechanisms of antibody-mediated AE and PNS mirror distinct clinical features, therapeutic approach and response, and long-term outcomes in these groups.

In antibody-mediated AE, the disease course is often stereotyped, as seen in anti-N-methyl-D-aspartate receptor (NMDAR) or anti-leucine-rich glioma inactivated 1 (LGI1) encephalitides, and specific clinical findings, such as facio-brachial dystonic seizures, may be pathognomonic.^75–77^

Furthermore, some autoantibodies, such as anti-immunoglobulin-like cell adhesion molecule 5 (IgLON5), may be present with a more chronic disease course and without overt signs of inflammation, mimicking neurodegenerative conditions rather than other causes of encephalitis.^78^

Treatment response is often favorable, and steroids, plasma exchange, IvIg, and rituximab are common therapeutic choices, given the B-cell-mediated pathogenesis. In about 10%–30% of patients, relapses can occur during the disease course and may resemble the first clinical event.^79–81^

In PNS, CNS involvement includes “high-risk” phenotypes such as limbic encephalitis, encephalomyelitis, opsoclonus-myoclonus syndrome, and rapidly progressive cerebellar syndrome. Different autoantibodies can present with the same clinical phenotype, and, in this setting, antibodies are useful markers for cancer, helping clinicians to identify the underlying malignancy with focused investigations.^82^ Recently, the introduction of immune checkpoint inhibitors has revolutionized cancer therapy and prognosis, but immune-mediated side disorders involving the nervous system have emerged as treatment side effects.^83^ Among neurological immune-related adverse events (n-irAEs), a subset of patients may have clinical features resembling classic PNS and have less frequent treatment response and a more unfavorable prognosis compared to other phenotypes.^84^

The identification and treatment of cancer is vital in PNS, as treatment outcomes are frequently unsatisfactory due to early neuronal death. Steroids and cyclophosphamide are usually preferred due to their rapid action, blood–brain barrier permeability, and depletion of both B and T cells. Relapses are rare but can occur as manifestations of cancer recurrence.^80^

Lastly, anti-GAD-65 neurologic autoimmunity encompasses autoimmune epilepsy, cerebellar ataxia, and stiff-person syndrome. The pathogenesis of GAD-65 autoimmunity is still not fully understood, and both humoral and cellular mechanisms may be involved. Response to treatment is variable across the spectrum, with stiff-person syndrome being the most responsive phenotype. Improvement can also be observed in patients with cerebellar ataxia, but seizure control in GAD-65 autoimmune epilepsy is often unsatisfactory.^85,86^

## Biomarkers in autoimmune encephalitides

Investigating markers of neuroglial damage can contribute to deciphering the pathogenesis, differential diagnosis, disease status, and prognosis of AE, with potential translation into clinical practice.

Many studies have demonstrated that neuroaxonal damage, reflected by increased concentrations of NfL in serum and CSF, occurs across different subtypes of AE, regardless of antibody specificity. These studies have mostly included patients with anti-NMDAR, anti-LGI1, anti-CASPR2, and anti-IgLON5 antibodies.^87–93^ Of note, NfL concentration can vary across different phenotypes associated with the same autoantibody, such as in GAD65 neurologic autoimmunity. In this case, patients with cerebellar ataxia and limbic encephalitis present higher concentrations of NfL than patients with stiff-person syndrome, implying different degrees of axonal damage across the spectrum.^94,95^ Similarly, patients with anti-NMDAR encephalitis usually have lower concentrations of NfL than those with other autoimmune encephalitides, mirroring the predominant synaptic dysfunction at disease onset in this condition.^88,89^

GFAP concentrations in AE may mirror astrocytic damage as seen in patients with anti-GFAP antibodies, an autoimmune astrocytopathy, who have CSF GFAP concentrations comparable to NMOSD with AQP4-Abs, reflecting a T-cell-mediated astrocytic injury.^96^ Alternatively, GFAP concentrations in AE may also indicate an astroglial reaction to inflammation, as hypothesized for anti-NMDAR encephalitis, in which serum GFAP concentration is increased only in the acute stage with a subsequent rapid decline and normalization after 6 months from onset.^97^ In addition, in patients with anti-IgLON5 disease, GFAP concentration is lower in those patients with subacute and pauci-symptomatic disease course.^93^

Neuronal damage also seems to occur in AE, as reflected by increased concentrations of total tau, phosphorylated tau, and 14-3-3 protein in some patients, usually to a lesser degree compared to neurodegenerative conditions.^98–101^ Furthermore, reduced amyloid beta concentration can also be observed, implying altered CSF dynamics and protein clearance due to neuroinflammation.^98^

Lastly, CSF markers of synaptic dysfunction, such as neurogranin, SNAP-25, beta-synuclein, and YKL-40, have been investigated with heterogeneous results. Some studies showed a reduction in synaptic marker concentration, implying antibody-mediated internalization, while others demonstrated increased concentration, suggesting their release after synaptic damage.^102,103^

The dynamics of these biomarkers over time has also important implications for the understanding of AE pathogenesis. The increase in NfL is delayed compared to the nadir of clinical disability and the peak of antibody titers, suggesting that neuroaxonal damage is not the major pathogenic cause of the disease, at least in anti-NMDAR encephalitis.^90,104^

The concentration of biomarkers is higher during the acute stages of the disease compared to remission, and, after the onset event, biomarkers tend to decrease over time, but may remain higher compared to reference values, suggesting residual neuroaxonal loss.^88,92,97,101,105^ Intriguingly, the dynamics of NfL concentration in patients with stable disease seems to be divergent in anti-NMDAR and anti-LGI1 encephalitides: indeed, NfL concentration tends to decline over time in anti-NMDAR encephalitis, while, in anti-LGI1 encephalitis, the decline is slower, and some patients may have fluctuating NfL concentrations during their follow-up.^92,97,105^ This different trend over time could be explained by frequent subclinical and potentially under-recognized disease activity, such as seizures occurring during sleep, recently demonstrated in anti-LGI1 encephalitis.^106^

Lastly, biomarkers tend to increase during relapses, although their concentration peaks tend to follow the clinical onset, mirroring what is observed at the onset event, but limiting the potential role of monitoring biomarkers over time to predict disease recurrence.^90,105^

Markers of neuroglial damage have a potential role in the differential diagnosis between AE and its mimics, although further prospective validation in clinical practice is mandatory before real-life implementation.

NfL evaluation may be useful to discriminate anti-NMDAR encephalitis from the relevant mimics, herpes simplex virus (HSV) 1 encephalitis, and the first episode of psychosis. Indeed, NfL concentration is higher in HSV 1 encephalitis and lower in psychosis without neurological accompaniments (primary psychiatric disease) compared to anti-NMDAR encephalitis. Of note, a cut-off concentration of serum NfL of 15 pg/mL allows to correctly identify 96% of patients with first episode psychosis and 85% of patients with AE.^87^ In addition, CSF NfL concentration is higher in neurodegenerative rapidly progressive dementia compared to anti-LGI1 encephalitis.^100^

Serum NfL concentration can also be useful to discriminate anti-IgLON5 disease from other bulbar mimics such as amyotrophic lateral sclerosis and myasthenia gravis,^107^ whereas CSF NfL concentration has moderate accuracy in differentiating autoimmune versus infectious inflammatory CNS disorders.^102^ In the same study, CSF markers of synaptic dysfunction, such as CSF beta-synuclein, SNAP25, and neurogranin, did not correctly discriminate infectious versus infective causes of encephalitis.^102^

Markers of neuronal damage have been investigated in the differential diagnosis of rapidly progressive dementias. Total tau can be increased in patients with AE,^93,98,99^ usually at a lower level compared to neurodegenerative conditions such as Alzheimer’s disease and Creutzfeldt-Jakob disease: for example, a cut-off of 100 pg/mL of total tau can be observed more frequently in patients with prion disease compared to autoimmune causes of dementia.^98^ Rarely, phosphorylated tau can be increased, and amyloid beta 42 can be decreased in patients with AE, but most patients do not have a CSF profile suggestive of Alzheimer’s disease.^93,98–100^ Lastly, 14-4-3 protein can be increased in patients with AE, especially in those harboring antibodies to GABA-B, and, in about 20% of cases, a CSF protein profile suggestive of CJD can be observed.^99^

The association between clinical features, disease outcomes, and markers of neuroglial damage has been investigated in different studies with divergent results. These discrepancies could be explained by heterogeneity in the spectrum of AE phenotypes and antibody specificities, the use of different outcome measures, variable sample timing from disease onset, or by differences in the analyzed matrix (i.e., serum, plasma, or CSF). Resolving this heterogeneity is crucial, as it could allow the development and translation into clinical practice of multimodal biomarkers (i.e., biological, neuroimaging, optical computerized tomography) to improve diagnostic accuracy and predict disease course, similarly to what is observed in demyelinating diseases.^108^

In anti-NMDAR encephalitis, NfL concentration is increased in patients with post-HSV-1 encephalitis or with coexistent demyelination or MRI abnormalities. CSF NfL concentration tracks with inflammatory CSF findings, movement disorders, seizures, and disease severity, while serum concentration seems to predict ICU admission. In the pediatric population, NfL predicts long-term outcomes and residual cognitive symptoms, while these findings are less consistent in adults, with divergent results regarding the correlation of long-term outcomes and NfL.^87,88,90,97,109^

In anti-LGI1 encephalitis, serum NfL seems to be associated with seizures and to predict long-term cognitive outcomes, while CSF concentration predicts the development of epilepsy.^91,92^

In anti-IgLON5 disease, serum NfL tracks with severity score at sampling, bulbar symptoms, and disability, while serum GFAP tracks with bulbar symptoms. In addition, serum NfL can predict treatment response and 1-year mortality.^93,107^

In anti-GFAP encephalomyelitis, CSF NfL correlates with poor outcome and the presence of brain MRI abnormalities.^96^

Lastly, studies investigating cohorts of patients with different antibody specificities and clinical syndromes have found heterogeneous correlations between concentration of serum or CSF total tau, GFAP, NfL, and markers of synaptic dysfunction with long-term clinical outcomes or with the development of hippocampal sclerosis.^89,95,101,102,110^ These divergent findings could be explained by the inclusion of patients with diseases that underlie heterogeneous pathogenic mechanisms and should be translated with caution in clinical practice.

## Biomarkers in PNS

As PNS are less frequently encountered compared to “idiopathic” AE cases, less evidence about the role of markers of neuroglial damage is available for this spectrum of disorders.

Some studies have highlighted that neuroaxonal loss is a major disease mechanism in the PNS, as serum NfL concentration is higher in patients with paraneoplastic neuronal intermediate filament autoimmunity, and CSF NfL concentration is increased in pediatric opsoclonus myoclonus syndrome compared to controls.^111,112^ In addition, patients harboring intracellular antigens or with an underlying malignancy tend to have higher CSF NfL concentration compared to their “non-paraneoplastic” counterparts.^113,114^

In patients with neurologic adverse events from immune checkpoint blockade, in particular those harboring anti-ANNA1/Hu antibodies, serum NfL is increased at comparable levels to HSV-1 encephalitis.^115^ Furthermore serum NfL concentration, but not GFAP, can discriminate patients with neurologic irAEs from other diseases. Similarly, markers of neuroglial damage are increased in patients with nervous system irAEs compared to immune-related hypophysitis and controls.^116^ NfL increase tend to be slower compared to other biomarkers such as S100B, but can predict treatment response and correlates with worst outcomes.^115–117^ NfL concentration is higher in patients with peripheral nerve involvement compared to neuromuscular toxicities, while GFAP concentration is more elevated in patients with CNS involvement.^116^

Lastly, longitudinal studies are currently lacking, but serum biomarker concentrations ((NfL, GFAP)) decrease over time and track with clinical status in some individual reports of patients with PNS or neurologic irAEs.^115,117,118–123^ Although promising in terms of potential clinical translations, the use of biomarkers to monitor these conditions is still in a preliminary phase, and longitudinal studies, also accounting for other factors such as chemotherapy-related neuroaxonal damage, are mandatory to draw definitive conclusions.

## Conclusion

In this review, we summarized the current evidence regarding the role of markers of neuroglial damage in autoimmune neurology.

Soluble biomarkers can aid our understanding of disease mechanisms and pathophysiology. Furthermore, these biomarkers can help clinicians in obtaining diagnostic precision by discriminating autoimmune disorders from their mimics, or by identifying specific biomarker signatures (i.e., astrocytopathy in NMOSD with AQP4-Abs). Lastly, these biomarkers can be helpful in predicting disease course and relapses, thus representing potential aid for treatment decisions.

Limitations of available studies hinder the translation of these findings into clinical practice and should be acknowledged. The heterogeneity of sampling timing, of assays for biomarker detection, and variability within included cohorts, especially for AE, are major confounders.

Future perspectives for clinical implementation of biomarkers include the standardization of assays used for their detection, the development and validation either of cut-offs or adjusted *z*-scores that can be translated from a study population to an individual patient level and are clinically significant (i.e., cut-offs defining impeding relapses) and, lastly, longitudinal studies to clarify the optimal time frame to assess biomarkers concentration at diagnosis and during follow-up.

**Figure 1.**
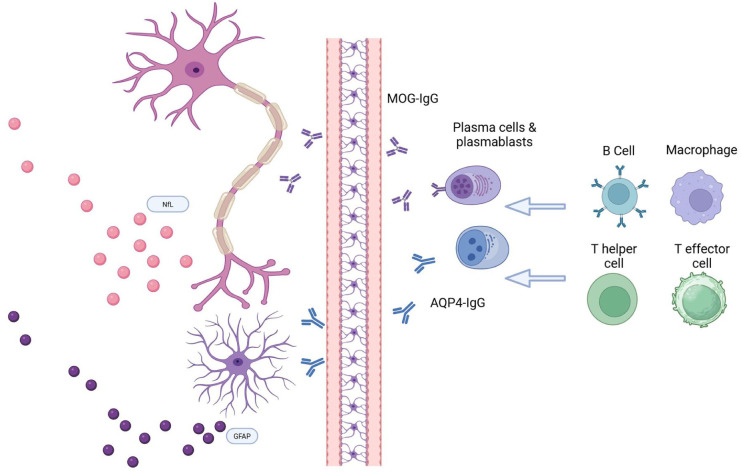
Immune pathogenesis and biomarkers of neuroglial damage in NMOSD and MOGAD. Source: Created with Biorender. AQP4-IgG, aquaporin-4 antibodies; MOGAD, myelin oligodendrocyte glycoprotein antibody-associated disease; MOG-Ig, myelin oligodendrocyte glycoprotein antibodies; NMOSD, neuromyelitis optica spectrum disorder.

**Figure 2.**
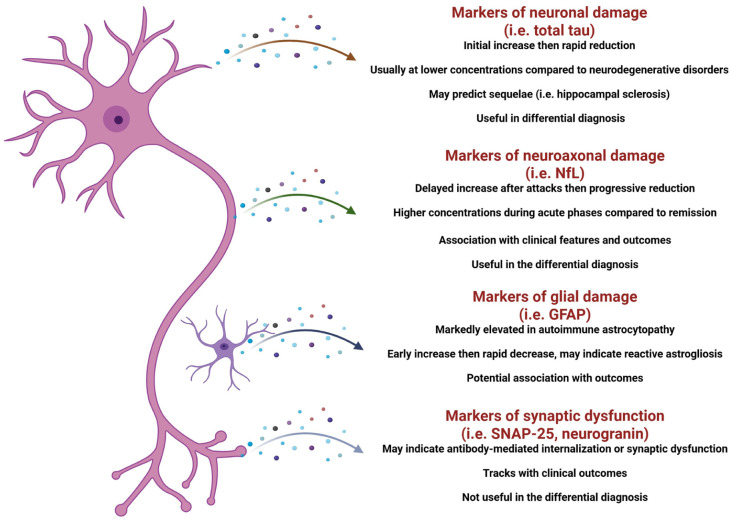
Summary of biomarkers of neuroglial damage investigated in patients with autoimmune encephalitis and paraneoplastic neurological syndromes. Source: Created with Biorender.

The inclusion of biomarkers in NMOSD clinical trials has greatly contributed to our understanding of their dynamics and potential role in clinical practice, highlighting the importance of conducting multicenter prospective studies for biomarker investigation and clinical practice translation, also in other autoimmune disorders of the nervous system (Figures 1 and 2).^57^

## Declarations

**Ethics approval and consent to participate:** Not applicable.

**Consent for publication:** Not applicable.

**Availability of data and materials:** Not applicable.
